# Diagnosis of Vertucci Type VII Anatomy in the Mandibular Left Lateral Incisor Using Digital Radiography: An Ex Vivo Case Study

**DOI:** 10.7759/cureus.63382

**Published:** 2024-06-28

**Authors:** Navdeep Jethi, Rachana Mishra, Rubaab Brar, Kanchan Bhadoria

**Affiliations:** 1 Conservative Dentistry and Endodontics, Daswani Dental College and Research Centre, Kota, IND; 2 Department of Pedodontics and Preventive Dentistry, Daswani Dental College and Research Centre, Kota, IND

**Keywords:** root canal anatomy, endodontic management, sectioning, radicular anatomy, mandibular lateral incisor, vertucci type vii, extralingual

## Abstract

Mandibular lateral incisors sometimes feature an additional lingual canal, which, if not identified and missed during endodontic treatment, can lead to postoperative pain. Thus, a proper diagnosis of the internal anatomy of a tooth is necessary before treatment begins. Radiovisiography (RVG), a cost-effective and widely employed dental imaging technique, is used in clinics to visualize the internal root structure of teeth. The zoom function of RVG allows for a detailed examination of complex internal anatomical variations, facilitating a more accurate diagnosis and treatment planning process. This case study identified a rare Vertucci Type VII root canal system in a mandibular left lateral incisor using the zoom-in feature of radiovisiography (Vatech, EZ sensor). Additionally, horizontal sectioning was performed, and these sections were imaged to verify the accuracy of digital radiographs in diagnosing such cases as part of a preclinical demonstration for aspiring endodontists.

## Introduction

Mandibular lateral incisors sometimes feature an additional lingual canal, which, when not identified, can lead to postoperative pain due to incomplete treatment [1,2]. Therefore, it is crucial to conduct a thorough diagnosis of the internal anatomy of a tooth before initiating treatment [1,2]. Also, the probability of finding two apical foramina in a single root of a lateral incisor is estimated to be 2.6% [3].

Type VII configuration in the Vertucci classification entails a single root canal that bifurcates into two canals, reunites into one, and subsequently divides into two distinct openings at the root apex, creating a unique root canal pattern (1-2-1-2) [2,3].

Radiovisiography (RVG), an essential and cost-effective dental imaging technique, is commonly utilized in clinics to visualize the intricate internal root structure of teeth [4,5]. The zoom function of RVG enables a detailed examination of complex internal anatomical variations, apical third anatomy, and the complex apical delta, thereby facilitating a more accurate diagnosis and treatment planning process [4-6]. Identifying an extra lingual canal using intraoral periapical radiographs (IOPA) and radiovisiography (RVG) can be influenced by the projection angle. Additional radiographs with different horizontal angulations (20°-30° mesial or distal) are needed to see the extra canals in the tooth separately, as regular angulation does not reveal them [4]. Mesial angulation may not completely reveal the true morphology of the canals [3]. In the context of in vivo root canal procedures, the utilization of cone beam computed tomography (CBCT) has been extensively discussed in numerous studies [3-5]. CBCT also has limitations in identifying small structures like calcified canals and lateral canals [4,5]. 

This case study revealed an exceptionally rare Vertucci Type VII root canal system in a mandibular left lateral incisor through the utilization of the zoom-in feature of radiovisiography (Vatech, EZ sensor), underscoring the critical role of detailed radiographic examination in endodontic diagnosis. This process was conducted as part of a preclinical demonstration for aspiring endodontists. Furthermore, horizontal sectioning was conducted to obtain images of the resulting sections, confirming the precision of digital radiographs in diagnosing such complex cases.

## Case presentation

**Figure 1:**
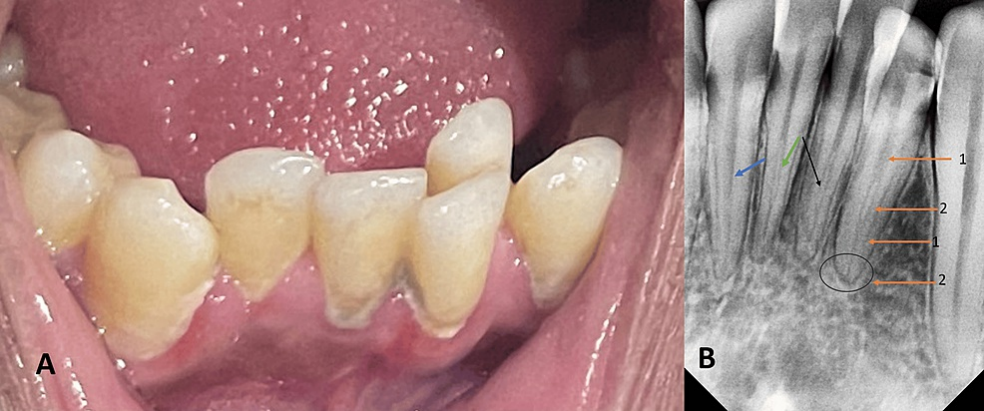
A) Lingually erupted left mandibular lateral incisor; B) Vertucci Type VII (1-2-1-2) root canal configuration in the left mandibular lateral incisor (blue circle and orange arrows); and C) Separate two root canals in the mandibular incisors (blue, green, and black arrows).

In this case study, preoperative pictures and radiographics were availed. The interpretation revealed that the lingually erupted left lateral incisor tooth number 32 had two separate canals with a Vertucci type VII root canal configuration (1-2-1-2). All the mandibular incisors exhibited an additional canal located outside the typical position, a rare and unique characteristic for this type of tooth (Figure 1).

**Figure 2:**
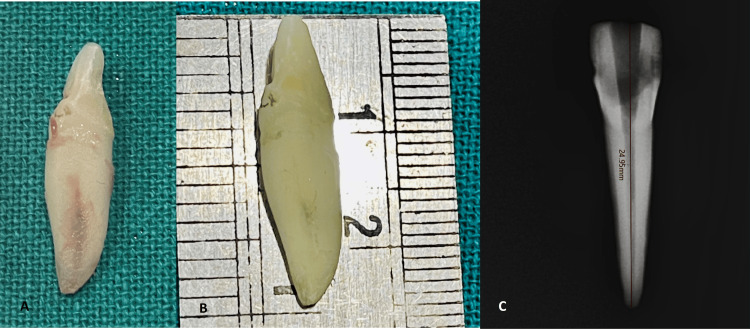
A) Extracted tooth 32; B) working length with metal scale (25 mm); C) working length with Ez sensor RVG software (24.95 mm)

Tooth number 32 was collected post-extraction from the oral surgery department. The tooth length, from the tip of the crown to the end of the root, including a 10 mm crown and a 15 mm root, was measured using a metal scale, resulting in a total length of 25 mm (Figure 2B). A prominent depression in the center of the root on the distal side created the illusion of two distinct roots coexisting within a single root structure.

**Figure 3:**
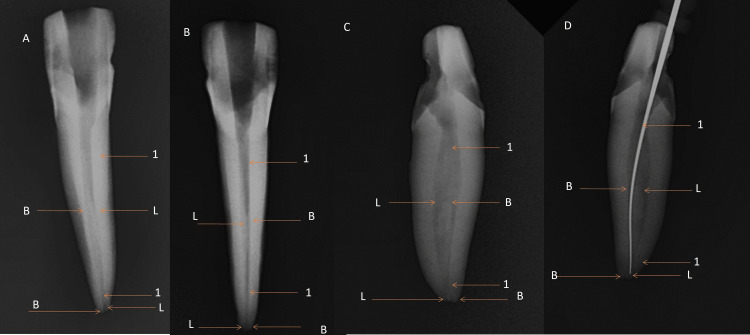
Multiangle radiographs for Vertuccii type VII (1-2-1-2) anatomy in 32 A) labial side; B) lingual side; C) mesial side; D) distal side L: lingual canal; B: buccal canal; *1: merging of two canals.

The access was opened using a round bur number 4 and further refined with an Endo Z bur (Dentsply, Bensheim, Germany). The remaining pulp in the tooth was removed from the pulp chamber, and a lingual extension of the access cavity in the cingulum area was made to locate the extra lingual canal. Meticulous removal of the roof of the cingulum area in a precise, ribbon-like manner from the buccal to the lingual aspect is crucial for accurately localizing the lingual canal during the procedure [7,8]. The tooth was radiographed from all sides using an RVG sensor (EZsensor Vatech). Mesial and distal angulation showed better visualization of two different buccal and lingual canals and the apical delta anatomy than the normal parallel technique (Figure 3). Digital radiographs confirmed the division of the pulp chamber into two distinct parts in the middle. 2-3 mm before the apex, these two canals unify to emerge as the separated apical openings of the buccal and lingual canals.

**Figure 4:**
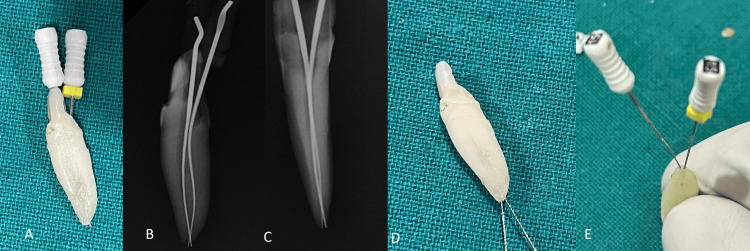
Confirmation of two apical canal openings for 32: A) tooth number 32 with two 15 K files; B) RVG mesial side; C) RVG labial side; D) and E) retrograde insertion of two 15 K files in buccal and lingual foramina

Two 15-k files (Mani, Utsunomiya, Japan) were inserted into both canals to check for patency and ensure thorough cleaning. Radiographs were then taken from different angles to confirm the exact canal configuration. The precise location of the two apical foramina was confirmed by retrogradely inserting the 15-k files at the root apices' openings, enabling a detailed assessment of canal ends and apical morphology (Figure 4).

Initially, the canal was prepared using files ranging from 15 to 25k lubricated with 17% EDTA, followed by enlarging the canal using rotary files (NeoEndo, Orikam Healthcare, Gurugram, Haryana, India) with an endomotor (Detsply). The irrigation protocol involved a combination of 2% NaOCl and normal saline to effectively alternate between tissue dissolution with NaOCl and tissue debris flushing with saline in the root canal system.

**Figure 5:**
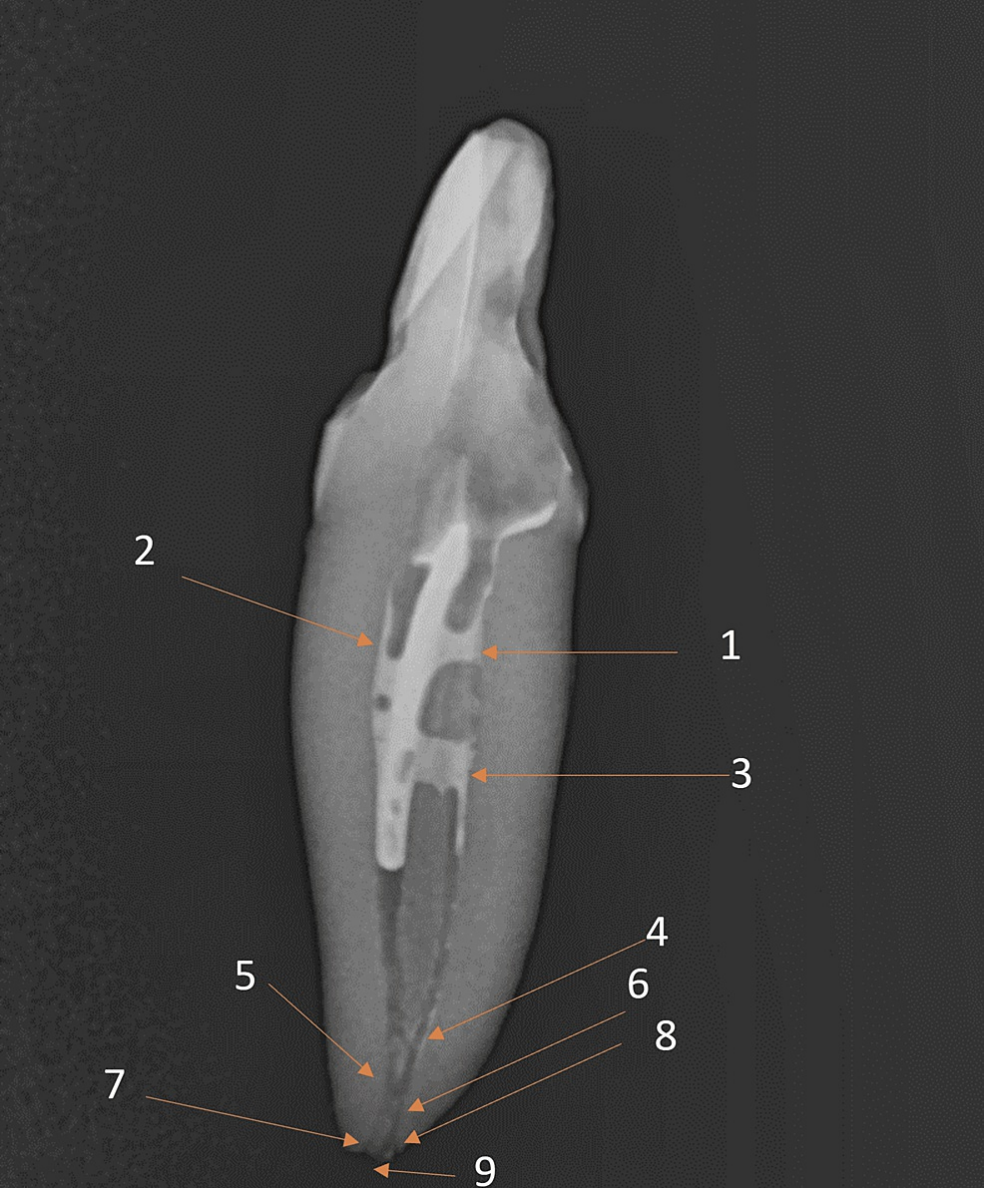
Detailed complex internal anatomy of the mandibular incisor: 1. Lingual canal 2. Buccal canal 3. Middle-third ramification 4. Apical ramification (A1); 5. Buccal and lingual canal unification 6. Apical ramification (A2) 7. Buccal apical opening 8. Lingual lateral canal opening 9. Lingual apical opening

**Figure 6:**
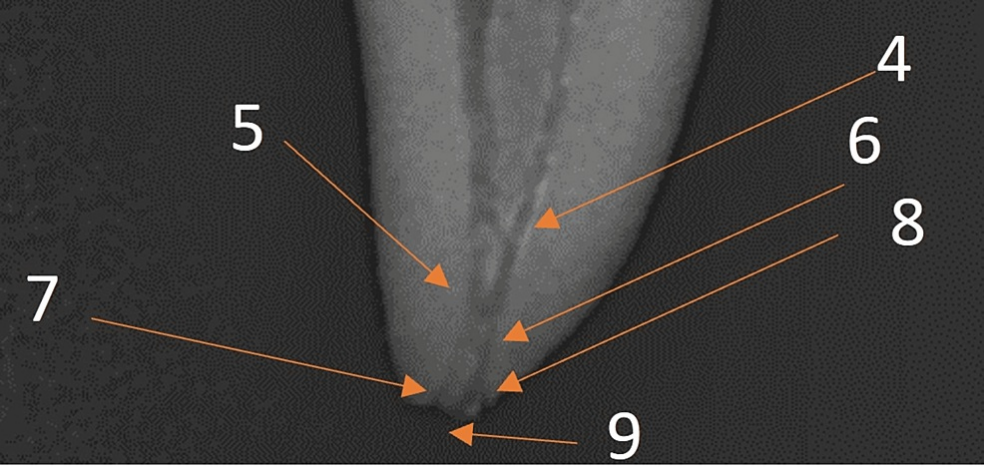
Detail apical anatomy 5. Buccal and lingual canal unification 6. Apical ramification (A2) 7. Buccal apical opening 8. Lingual lateral canal opening 9. Lingual apical opening

The apical delta, a significant anatomical feature representing a branching structure at the apex, was clearly visible on the radiographs, emphasizing important details in the apical third of the root. An apical ramification (A1) occurs before the buccal and lingual canals unify in the apical third of the root. A second apical ramification (A2) is where two canals merge to separate back in two apical openings, buccal and lingual canal openings. There was a lateral canal opening beside the lingual canal apex (Figures 5, 6).

**Figure 7:**
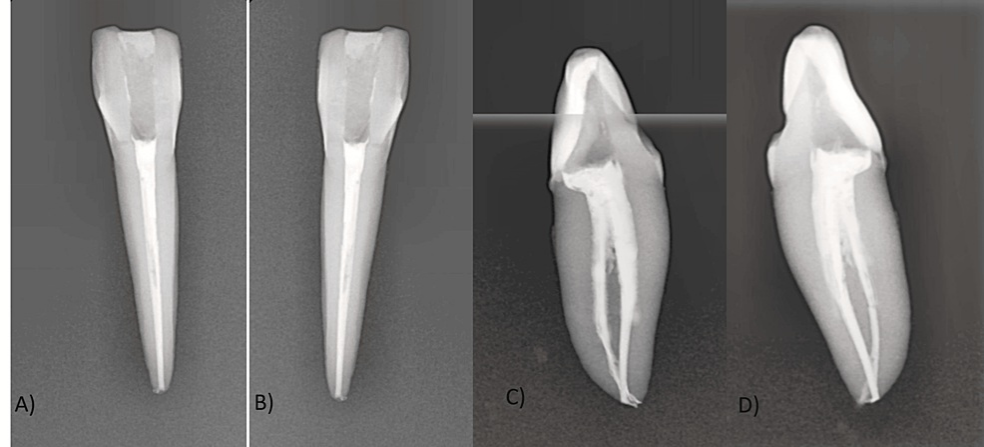
Obturation from different angles A) labial view; B) lingual view; C) mesial view; D) distal view

During the master cone try, the buccal canal was obstructing the lingual and its lateral canal at the apex. The canals were dried by using paper points and removing excess moisture with syringe aspiration from the canals. Obturation was performed using F1 4% taper gutta-percha points and AH Plus resin-based sealer (Figure 7). The lingual and its lateral canal remained I mm short of the apex.

The post-endodontic restoration involved using a combination of flowable and restorative composites for optimal results.

**Figure 8:**
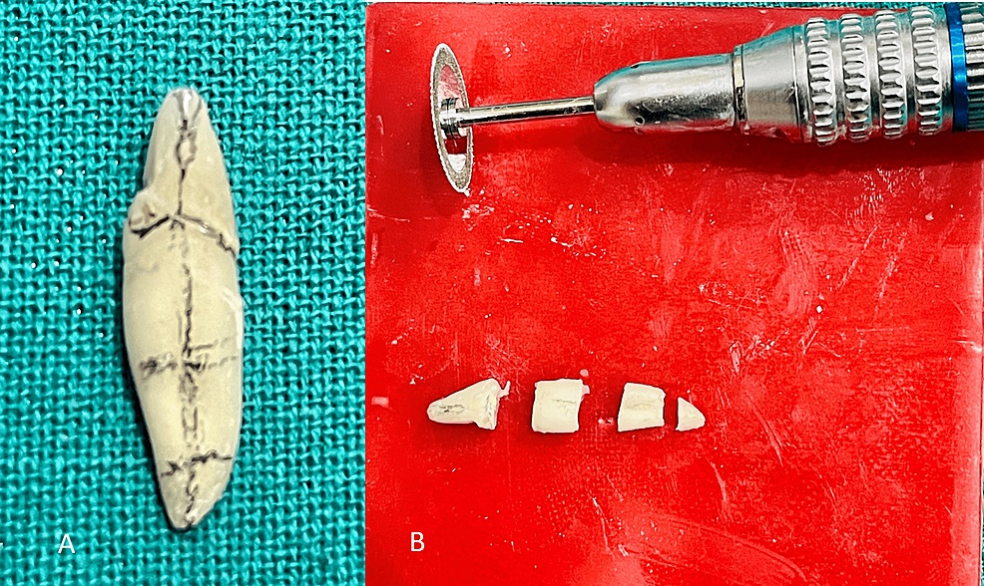
Dividing the tooth into four parts using a sectioning disc: A) Marking the tooth for sectioning B) Horizontal sections of the tooth crown part and radicular part (coronal third, middle third, and apical third)

Horizontal sectioning using a round-cut sectioning disc on a micromotor straight handpiece involved marking the tooth at the coronal cervix and dividing it into three sections for the root apical portions, as shown in Figures 8A, 8B.The tooth was segmented into four distinct parts: the crown portion at the cervical margin, the coronal third of the root, the middle third of the root, and the apical third of the root, as shown in Figure 8B.

**Figure 9:**
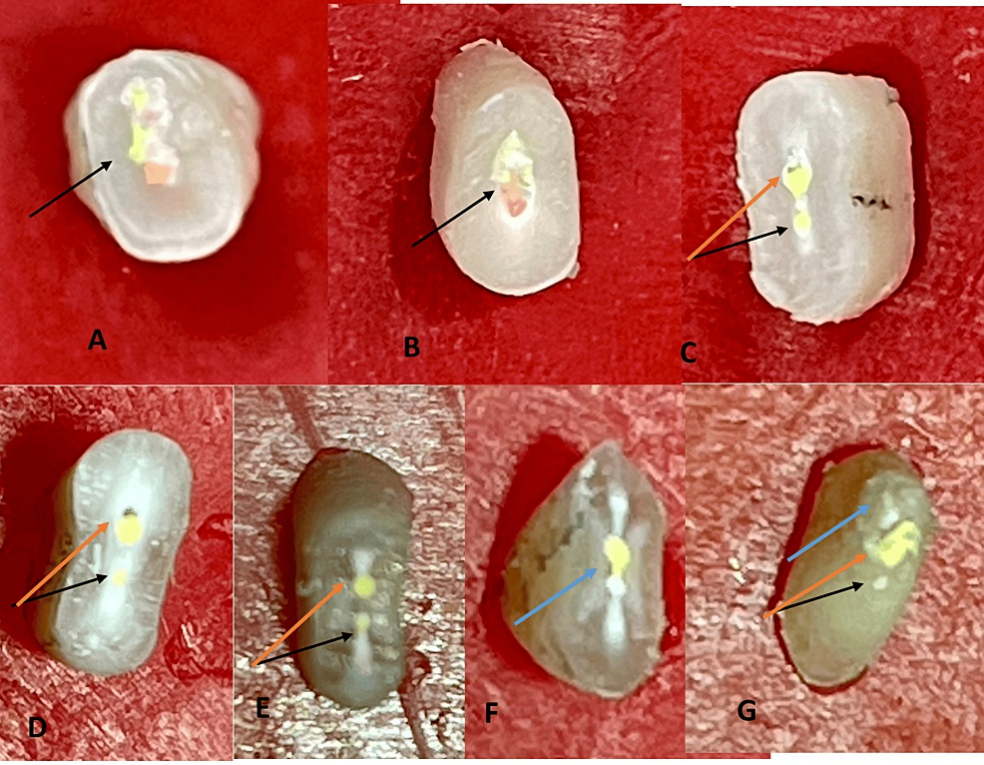
Vertucci type VII anatomy in mandibular lateral incisor A) single opening in the pulp chamber B) a single canal in the superior coronal part (black arrow); C) an inferior coronal root section separates the buccal canal (orange arrow) and lingual canal (black arrow); D) a superior middle root section separates the buccal canal (orange arrow) and lingual canal (black arrow). E) inferior middle root section: separated buccal canal (orange arrow) and lingual canal (black arrow); F) superior apical root section: buccal and lingual canal merging into one, G) Root apex having two separate apical openings, buccal (black arrow) and lingual (orange arrow); lingual lateral canal opening (blue arrow)

Each root section was meticulously imaged using an 11X zoom on an iPhone 12 Pro camera to guarantee high-definition viewing and clarity of the internal structures (Figure 9). These pictures were examined to facilitate visualization of the horizontal sections from both superior and inferior perspectives, aiding in a comprehensive analysis of the tooth's internal structure. An enlarged opening (Figure 9A) on the lower side of the crown portion extended into the upper section of the root coronal third, indicating a structural connection (Figure 9B). Further observations revealed two distinct filling points for the lingual and buccal canals at the lower part of this section, where the significant distal depression of the root begins (Figure 9C). In the root middle third section, the greater distance between the buccal and lingual canals in the upper part compared to the lower part indicated a notable anatomical variation (Figure 9 D-E). In the apical third section of the root, both canals appeared to merge into a single canal, distinctly separated only by the resin sealer, without any intervening dentinal structure present (Figure 9F). On the lower side of this root apex section, two separate orifices, representing the buccal and lingual apical openings, were seen, along with a lateral canal to the lingual (Figure 9G).

## Discussion

In this case study, a rare Vertucci type VII (1-2-1-2) root canal configuration in mandibular lateral incisors was observed with RVG zoom and in magnified horizontal sections. In such cases, the extra-lingual canal often goes undiagnosed because a single canal opening in the pulp chamber bifurcates in the middle third of the root [2,3]. An undiagnosed extra-lingual canal in the mandibular incisors can lead to post-obturation pain, root canal infections, and compromised treatment outcomes, ultimately jeopardizing the success of the dental procedure. [1,4,6,7,9].

Recognizing the rarity and distribution of Vertucci type VII internal anatomy is essential for precise diagnosis and treatment planning in endodontics. Han et al. (2014) and Valenti-Obino F. et al. (2019) reported that Vertucci type VII internal anatomy is rare, found in 0.4% and 0.08% of Chinese and Italian mandibular lateral incisors, respectively [9-11].

The Indian population shows the highest prevalence of a lingual canal, indicating that racial and ethnic factors influence its occurrence [1,2]. The occurrence of an extra-lingual canal in mandibular lateral incisors was reported to be 36% in the North Indian population by Boruah LC (2014) and 26% worldwide by Martin et al. (2023) [12,13].

Our study uncovered another notable and uncommon discovery: two separate apical openings at the apex of the single-rooted lateral incisor, highlighting the complexity of dental anatomy and its impact on endodontic procedures. According to a study by Boruah LC (2014) in North Indian populations, the prevalence of having two separate apical foramina for a mandibular incisor is reported to be 6.25% [12]. In previous in vivo case reports, it was observed that the double canals of the mandibular lateral incisors were similar to ours [7-9]. Also, in these case reports, both canals typically converge into a single apical opening [6-8]. In the present case, contrary to expectations, two different apical openings (buccal and lingual) with a lateral canal attached to the lingual canal at the apex were found in radiographs as well as in a horizontal section.

The internal anatomy of a tooth can be examined using a range of radiography techniques, such as 2D RVG and advanced methods like CBCT, which provide detailed 3D visualizations for accurate diagnoses [4-6]. Apart from RVG, micro-computed tomography is another technique that can be used for ex vivo studies [5]. In this ex vivo case study, the tooth was radiographed using RVG from buccal, lingual, mesial, and distal aspects. Findings include overlap between the two canals in buccal and lingual aspects, and more accurately, no overlapping of the canals and microanatomy in the mesial and distal aspects of radiographs using the zoom-in feature of the RVG (EZ sensor). This can be related to the 20°-30° mesial or distal X-ray exposure angle being more accurate in capturing the internal anatomy of this tooth as compared to the normal paralleling angle technique [3,4].

In mesial angle radiographs, the tooth was radiographed after the application of the radioopaque sealer on the walls of the canals, and this radioopaque sealer made the canals more visible on RVG [3]. In mesial and distal aspect radiographs, the ramifications are intricately positioned 3-4 mm away from the apex. They demonstrate the convergence of the lingual and buccal canals, creating two distinct apical foramina. This complex apical delta has two ramifications and a lateral canal to the lingual canal, which has been said to have a 7% chance in the lateral incisor [2,3].

In the absence of radiographs, the dentinal map plays a crucial role in diagnosing a missed canal [3]. The presence of two root canals in the mandibular lateral incisors frequently corresponds with buccolingual dentinal maps [2-4]. In the Vertucci Type VII (1-2-1-2) configuration, the dentinal map bifurcates in the middle of the root, demonstrating a single canal that bifurcates at the end of the coronal third [2], which makes the dentinal maps confusing to read [2].

The canals were obturated to assess the true trajectory of the canals and validate the accuracy of the radiographic images. This obturated tooth was horizontally sectioned to compare the virtual reality of radiographs on screen with the actual trajectory of the filling materials. Horizontal sections were imaged and zoomed in with an 11x zoom on an iPhone 12 Pro Max to examine both the upper and lower sides in this study. Magnified images are highly effective in showing the number and locations of apical foramina by providing superior visibility and detail [5,14].

The accurate alignment between the virtual on-screen diagnosis of root canals and the horizontal section of obturated canals confirms the presence of the Vertucci type VII configuration (1-2-1-2) [14].

## Conclusions

When it comes to single-rooted mandibular lateral incisors, the root canal configuration called Vertucci type VII (1-2-1-2) is not common. This is because the single canal splits into two branches in the middle third of the root, which makes it hard to find the extra lingual canals. Failure to diagnose such extra lingual canals with intricate apical deltas in mandibular incisors can lead to post-obturation pain and pathological conditions. Clinicians must carefully study digital X-rays by utilizing the RVG zoom function to accurately locate the internal structure of the roots during the treatment of mandibular incisors. Utilizing the RVG zoom feature enhances the visualization of the apical deltas and lateral or accessory canals, leading to improved treatment outcomes.
